# A220 LONG-TERM EFFECTIVENESS AND SAFETY OF USTEKINUMAB DOSE ESCALATION IN PATIENTS WITH REFRACTORY ULCERATIVE COLITIS: A MULTICENTER RETROSPECTIVE COHORT STUDY

**DOI:** 10.1093/jcag/gwae059.220

**Published:** 2025-02-10

**Authors:** L Albino, L van Lierop, R Rosentreter, P Thomas, C Lu, J Siffledeen, K Kroeker, C Ma, F Peerani, B Halloran, D C Baumgart, L Dieleman, L Du, F Hoentjen, K Wong

**Affiliations:** University of Alberta Department of Medicine, Edmonton, AB, Canada; University of Alberta Department of Medicine, Edmonton, AB, Canada; University of Calgary Cumming School of Medicine, Calgary, AB, Canada; Radboudumc Afdeling Maag- Darm- en Leverziekten, Nijmegen, Gelderland, Netherlands; University of Calgary Cumming School of Medicine, Calgary, AB, Canada; University of Alberta Faculty of Medicine and Dentistry, Edmonton, AB, Canada; University of Alberta Division of Gastroenterology, Edmonton, AB, Canada; University of Calgary Cumming School of Medicine, Calgary, AB, Canada; University of Alberta Faculty of Medicine and Dentistry, Edmonton, AB, Canada; University of Alberta Division of Gastroenterology, Edmonton, AB, Canada; University of Alberta Division of Gastroenterology, Edmonton, AB, Canada; University of Alberta Department of Medicine, Edmonton, AB, Canada; University of Alberta Department of Medicine, Edmonton, AB, Canada; University of Alberta Division of Gastroenterology, Edmonton, AB, Canada; University of Alberta Division of Gastroenterology, Edmonton, AB, Canada

## Abstract

**Background:**

Ustekinumab dose escalation (DE) may be an effective strategy to recapture clinical response in patients with ulcerative colitis (UC).

**Aims:**

The aim of this study was to assess the real-world long-term effectiveness and safety of ustekinumab DE in patients with refractory UC.

**Methods:**

This multicenter retrospective cohort study included patients with refractory UC who received at least one IV induction ustekinumab dose between January 2016 and November 2021. We compared ustekinumab DE to no DE, examining clinical, biochemical, and endoscopic disease outcomes. The primary endpoint was corticosteroid-free clinical remission (partial Mayo score ≤ 2 without systemic corticosteroids) at the end of follow-up. Cox-proportional hazards regression analysis was performed for factors associated with time to DE and a Kaplan-Meier plot was created for visualizing drug persistence probabilities.

**Results:**

We enrolled 121 patients. Eighty-one patients (67%) underwent DE during a median follow-up of 141 weeks. Corticosteroid-free clinical remission at the end of follow-up was achieved for 53.2% (DE group) and 59.0% (non-DE group). In the DE group, 53% discontinued ustekinumab, mainly due to a lack of effectiveness. At the end of follow-up, 47% of DE patients remained on ustekinumab, compared to 55% in the non-DE group. Ustekinumab persistence probability after 2 years was 40% (DE group) versus 79% (non-DE group). Only 2 patients discontinued ustekinumab for adverse events.

**Conclusions:**

Our results indicate that DE is a common method for optimizing ustekinumab treatment in refractory UC. While DE appears safe, effectiveness and drug persistence of DE beyond 2 years are limited.

Table 1

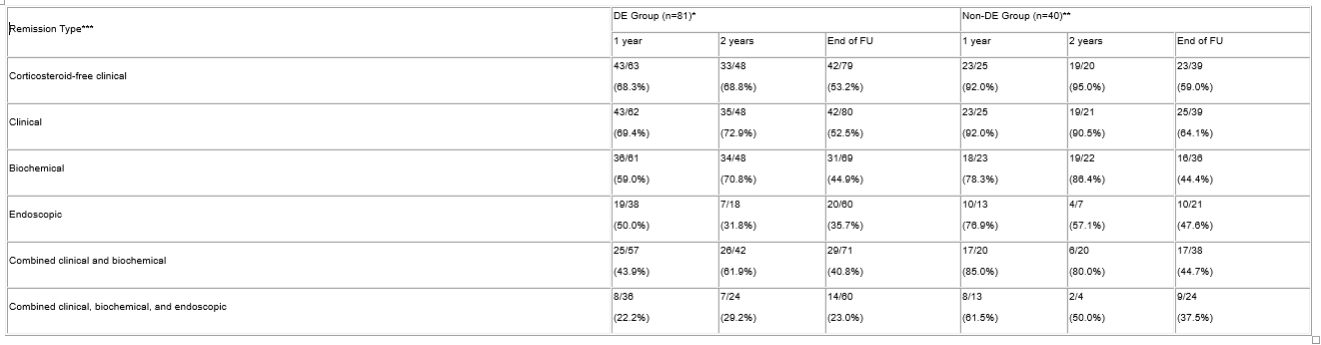

Proportion of participants in corticosteroid-free clinical, biochemical, combined clinical/biochemical, endoscopic, and combined clinical/biochemical/endoscopic remission at year 1, year 2, and end of follow up for the DE group vs non-DE group. *In the DE group, 8 discontinued ustekinumab before 1 year and an additional 14 before 2 years. **In the non-DE group, 12 discontinued ustekinumab before 1 year and an additional 4 before 2 years. ***Additional patients missing from the denominators had missing values at the presented timepoints.

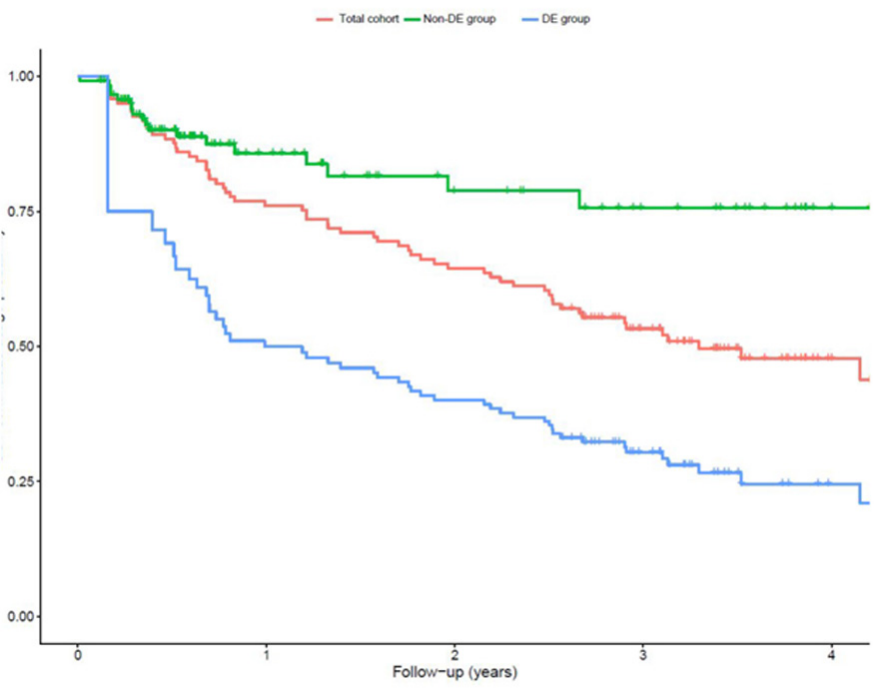

Kaplan-Meier plot for probabilities of continued ustekinumab usage over the duration of follow-up in years. The green (upper) line represents the non-DE group, the red (middle) line represents the entire cohort, and the blue (bottom) line represents the DE group.

**Funding Agencies:**

None

